# Physical Activity and Sedentary Patterns among Metabolically Healthy Individuals Living with Obesity

**DOI:** 10.1155/2018/7496768

**Published:** 2018-03-08

**Authors:** Marika de Winter, Brittany V. Rioux, Jonathan G. Boudreau, Danielle R. Bouchard, Martin Sénéchal

**Affiliations:** ^1^Cardio-metabolic Exercise & Lifestyle Lab, 2 Peter Kelly Drive, P.O. Box 4400, Fredericton, NB, Canada; ^2^University of New Brunswick, Faculty of Kinesiology, 2 Peter Kelly Drive, P.O. Box 4400, Fredericton, NB, Canada; ^3^New Brunswick Institute for Research, Data & Training, University of New Brunswick, 38 Dineen Drive, 304F Keirstead Hall, Fredericton, NB, Canada

## Abstract

**Background:**

Some individuals living with obesity are free from typical cardiometabolic risk factors and are termed metabolically healthy obese (MHO). The patterns of physical activity and sedentary behaviors among MHO are currently unknown.

**Methods:**

This study includes 414 youth (12–18 years old), 802 adults (19–44 years old), and 1230 older adults (45–85 years old) living with obesity from the 2003-2004 or 2005-2006 NHANES cycles. Time spent in bouts of 1, 5, 10, 30, and 60 minutes for moderate-to-vigorous physical activity (MVPA) and sedentary time was measured objectively using accelerometers. Participants were categorized as MHO if they had no cardiometabolic risk factors above the identified thresholds (triglycerides, high-density lipoprotein cholesterol, systolic blood pressure, diastolic blood pressure, and glucose).

**Results:**

The proportion of MHO was 19%, 14%, and 12% in youth, adults, and older adults, respectively. MHO adults displayed a higher 1-minute bout of MVPA per day compared to non-MHO (*p* = 0.02), but no difference was observed for MVPA and sedentary behavior patterns for youth and older adults. When adjusted for confounders, all bouts of sedentary behavior patterns in youth were significantly associated with being classified as MHO.

**Conclusion:**

This study suggests that greater sedentary time is associated with cardiometabolic risk factors in youth even if they are physically active.

## 1. Introduction

The growing prevalence of obesity raises concerns as this condition is associated with cardiometabolic risk factors including high blood pressure, hyperlipidemia, insulin resistance, and type 2 diabetes (T2D) [1, 2]. However, a proportion of individuals living with obesity are free of typical cardiometabolic risk factors [3] and are at lower risk of T2D. This subgroup of the population is termed metabolic healthy obese (MHO) and varies between 7–74% in youth and adults depending on the definition [4–6]. Despite high adiposity, MHO individuals display a favorable metabolic profile characterized by a high insulin sensitivity, lack of hypertension, favorable lipid profile, satisfactory body fat distribution, and a low level of systemic inflammation [7].

A body of evidence suggests that physical activity is positively associated with better cardiometabolic risk factor profiles [8–12]. For example, Prince et al. (2014) [11] found that moderate-to-vigorous physical activity (MVPA) was the strongest predictor of being MHO in youth; however, this association was not statistically significant when using another definition of MHO. Although self-reported measures of physical activity were used in this study, they suggest the need to confirm the association between physical activity and using an objective measure of physical activity. On the other hand, time spent sitting or in another sedentary behavior has also been negatively associated with being MHO [12–16]. For example, a recent study performed with 107 adults reported that MHO individuals spent less time in sedentary behavior compared to non-MHO (563.5 versus 593.0 mins., *p* = 0.02), but nothing was reported on sedentary beheviour or physical activity patterns [12].

Physical activity patterns are usually defined in bouts of physical activity at different intensities. Most national and international agencies recommend that adults and older adults perform a certain amount of minutes of physical activity (typically 150 minutes) at moderate-to-vigorous intensity in 10-minute bouts or more [17, 18]. However, since it is possible to quantify short bouts of physical activity using devices such as accelerometers, many have reported that any bout of physical activity spent at moderate or vigorous intensity would be associated with health benefits [19–23]. It is currently unknown what these patterns are for MHO and if they differ from non-MHO individuals. In addition, it is unknown whether patterns of MVPA are influenced by patterns of sedentary behavior. Moreover, previous studies failed to (1) use objective measures of physical activity, (2) quantify physical activity and sedentary behavior patterns, and (3) and investigate different age groups among MHO and non-MHO individuals [11, 12, 24].

To address this gap in the literature, we performed a cross-sectional study to investigate physical activity patterns of MVPA and sedentary behavior patterns among MHO and non-MHO individuals in a wide spectrum of ages using the *National Health and Nutrition Examination Survey* (NHANES). The objective of this study was to investigate the association between sedentary behavior and MVPA patterns using an objective measure of physical activity and the likelihood of being MHO. The second objective of this study was to investigate whether MHO individuals have different sedentary behavior and MVPA duration patterns compared to non-MHO individuals. Our hypothesis was that sedentary and MVPA duration patterns would be associated with being MHO across all age groups. Our second hypothesis was that MHO individuals would be characterized by a longer duration pattern of MVPA (more total amount of MVPA performed in bouts) and a shorter duration pattern of sedentary behavior (less total amount of sedentary time performed in bouts) compared to non-MHO individuals.

## 2. Methods

### 2.1. Study Population

This study sample consisted of 2446 participants, whom participated in the 2003-2004 or 2005-2006 *National Health and Nutrition Examination Survey* (NHANES). Participants included 414 youth (aged 12–18), 802 adults (aged 19–44), and 1230 older adults (aged 45–85). From the original 2003-2004 and 2005-2006 NHANES, 9750 participants wore an accelerometer and have available accelerometer data. From these participants, 7304 were excluded for the following reasons: (1) did not have at least four valid days of accelerometer wear time, (2) were less than 12 years of age and therefore have no age- and sex-specific cut-points for cardiometabolic risk factors used to categorize youth as MHO or non-MHO, (3) did not have a BMI ≥ 30 kg/m^2^ for adults or the 95th percentile for youth, or (4) had missing data for at least one cardiometabolic risk factor used for the categorization of MHO. Participants included in this analysis had similar general characteristics compared to those excluded from the analysis, including age (youth = 14.8 ± 1.9 versus 14.8 ± 2.0; adults = 31.3 ± 7.8 versus 32.7 ± 7.5; older adults = 63.9 ± 12.0 versus 62.0 ± 10.8 years) and the proportion of male to female participants (youth = 51.5% versus 50.7%; adults = 48.4% versus 44.6%; older adults = 49.9% versus 44.0%).

In NHANES, subjects were identified using a stratified multistage probability sampling design. Detailed survey operation manuals and consent forms are available on the NHANES website [25]. Briefly, the NHANES survey consisted of a home interview and a thorough health examination. During the interview, participants were asked questions about their health status, ethnicity, chronic condition history, and lifestyle behaviors. The health examination was performed in a mobile examination center. All participants provided written and informed consent. The *National Center for Health Statistics* approved the protocol.

### 2.2. Anthropometrics

Height (cm), weight (kg), and waist circumference (cm) were measured by an NHANES examiner. Thereafter, body mass index (BMI) was calculated using the following formula [weight (kg)/height (m^2^)]. The BMI *z*-score was calculated using the *Centers for Disease Control and Prevention* reference chart [26]. The protocol was based on the age of the participants at the time the measurements were taken. The measurement procedures for each variable can be found in the *Anthropometric Standardization Reference Manual* [27].

### 2.3. Primary Outcome Variable

#### 2.3.1. Metabolically Healthy Obese

There are several definitions for classifying individuals as MHO [6]. Each definition includes various criteria and cut-offs which leads to a wide variability in research findings [6]. Some definitions include “normal” insulin sensitivity, “low” insulin resistance (measured by homeostatic model assessment), and the absence of any cardiometabolic risk factors [6]. To facilitate comparisons with other studies, we classified participants as MHO if they had a BMI ≥ 95th percentile for youth and a BMI ≥ 30 kg/m^2^ for adults and older adults and did not present a clinically relevant elevation in any of the following cardiometabolic risk factors: plasma triglycerides, plasma glucose, systolic blood pressure, diastolic blood pressure, or an abnormally low high-density lipoprotein cholesterol (HDL-c). The age- and gender-specific cut-points used for the classification of youth have been published elsewhere [28] and previously used by our group [3]. For adults and older adults, the cut-points used were obtained from the harmonized definition of metabolic syndrome proposed in 2009 [29]. MHO in adults and older adults were defined as the absence of the following cardiometabolic risk factors: triglycerides < 1.7 mmol/L; HDL-c men ≥ 1.0 mmol/L, women ≥ 1.3 mmol/L; systolic and diastolic blood pressure < 130/85 mmHg; and fasting blood glucose < 5.6 mmol/L. In all age groups, MHO was defined as follows: MHO: 0 cardiometabolic risk factors; non-MHO: ≥1 cardiometabolic risk factors. Considering the fact that a strong correlation between waist circumference and BMI (*r* = 0.89; *p* = 0.0001) was observed, the waist circumference was not considered in the criteria, which would have made the MHO and non-MHO categorization more challenging.

#### 2.3.2. Cardiometabolic Risk Factors

Plasma HDL-c, triglyceride, and glucose concentrations were measured at a fasting state [30, 31]. Systolic and diastolic blood pressures were measured at rest according to the NHANES procedure [32]. The average of the first two measurements were used as the blood pressure variable.

### 2.4. Primary Exposure Variable

#### 2.4.1. Physical Activity Patterns

Participants wore an accelerometer (actigraph) on their right hip on an elasticized belt that could be customized to each participants' waist circumference. Participants wore the accelerometer during waking hours for seven consecutive days. The data was considered valid if the device had been worn for a minimum of four days (independently of the day of the week—weekday or weekend) with 10 hours of wear time per day [33, 34]. Wear time was defined as subtracting nonwear time from 24 hours. Nonwear time was identified by at least 60 consecutive minutes of counts between 0 and 100. Participants were instructed to remove the devices only when in water (swimming or bathing) and when sleeping. Age-specific cut-points were used to quantify sedentary, light, moderate, and vigorous PA [35–37]. To investigate the impact of physical activity patterns on being considered MHO, different lengths of physical activity bouts were examined: 1, 5, 10, 30, and 60 minutes of MVPA and sedentary time were established.

#### 2.4.2. Covariates

Covariates included in the analyses were age, gender, race/ethnicity (non-Hispanic white, non-Hispanic black, Hispanic, and other), education level (less than high school, high school, college, or university), and family income (<$20,000, $20,000–75,000, or ≥$75,000), which were all self-reported during the interview at the mobile examination center.

### 2.5. Statistical Analysis

Data are presented as means ± standard deviations for continuous variables while categorical variables are presented as *N* (%). Independent *t*-tests were used to quantify differences between non-MHO and MHO. Survey-weighted logistic regressions were used to identify whether physical activity patterns were independent predictors of being MHO. All analyses used survey weights to account for the complex sample and survey designs. Odds ratios are expressed in hours. The youth models were adjusted for BMI *z*-score, age, sex, time (1-min. bouts) spent in a sedentary state, ethnicity, and family income categories. The adult and older adult models were adjusted for BMI, age, sex, time (1-min. bouts) spent in a sedentary state, ethnicity, education level, and family income categories. Data management and analysis were performed using SAS version 9.4 of the SAS System for Windows. A *p* < 0.05 was considered significant.

## 3. Results

### 3.1. General Characteristics

The prevalence of MHO was 19% in youth, 14% in adults, and 12% in older adults (Table 1). Regardless of the age group, no significant differences were observed between MHO and non-MHO in terms of general characteristics. As expected, differences were observed between MHO and non-MHO for most of the cardiometabolic risk factors variables. In youth, MHO had better cardiometabolic risk factors compared to non-MHO for HDL-c, triglycerides, and plasma glucose (*p* < 0.05). In adults and older adults MHO, all the cardiometabolic risk factors were significantly better compared to non-MHO (*p* < 0.05), with the exception of diastolic blood pressure.

### 3.2. Difference in Sedentary Behavior and MVPA Patterns among MHO and non-MHO

No differences were observed in sedentary behavior patterns between non-MHO and MHO among the three age groups (Table 2). However, differences were observed in MVPA patterns, but only in the adults group. MHO adults spent more time in 1-minute, 5-minute, and 10-minute bouts of MVPA (all *p* < 0.05).

### 3.3. Association between MVPA Bouts and MHO Individuals

Table 3 describes the proportions of individuals in each age group with 1-minute, 5-minute, 10-minute, 30-minute, and 60-minute bouts of MVPA. All individuals in the sample accumulated MVPA time in 1-minute bouts. However, that proportion dropped abruptly as the bout length increased, and less than 1% of the sample achieved 60-minute bouts of MVPA in any age group. After adjusting for confounders, including total time spent in sedentary behavior (1-minute bouts), MVPA patterns were not associated with being MHO (youth: OR 0.810, 95% CI 0.338–1.942; adults: OR 1.653, 95% CI 0.891–3.068; older adults: OR 1.815, 95% CI 0.900–1.152; all *p* > 0.05).

### 3.4. Association between Sedentary Bouts and MHO Individuals

The proportions of individuals in each age group with 1-minute, 5-minute, 10-minute, 30-minute, and 60-minute bouts of sedentary behavior patterns were calculated (Table 3). More than 99% of the sample, in all age groups, spent time in 1-minute, 5-minute, 10-minute, and 30-minute bouts of sedentary behavior during the evaluated week. Respectively, 83.0%, 87.3%, and 93.1% of youth, adults, and older adults spent time in 60-minute bouts of sedentary behavior during that same week. After adjusting for confounders, including total time spent in MVPA, sedentary behavior was associated with being MHO for youth (OR 0.677; 95% CI 0.479–0.958; *p* = 0.030) regardless of the length of sedentary bouts. Sedentary behavior patterns were not associated with being MHO for adults and older adults after adjustment for confounders (OR 0.968; 95% CI 0.828–1.131; *p* = 0.678).

## 4. Discussion

The first objective of this study was to investigate the associations between physical activity patterns, sedentary behavior patterns, and the likelihood of being MHO across a broad spectrum of ages. Our findings show that only sedentary behavior patterns in youth living with obesity were significantly associated with being classified as MHO. This result was significant even when controlling for potential confounders, including total time spent at MVPA. Given the fact that increasing long-term physical activity levels in children normally fails, these results suggest that reducing sedentary behavior in youth living with obesity might reduce the odds of cardiometabolic risk factors associated with obesity and T2D.

Previous studies have shown that physical activity spent at moderate intensity was protective of cardiometabolic risk factors in individuals living with obesity [19–23]. This study confirms these results. In fact, MVPA was significantly associated with being considered MHO before adjusting for sedentary behavior. However, our results do not support an association between MVPA and the likelihood of being MHO in any of the age groups when adjusted for sedentary time. The discrepancy between our results and those from the literature might be explained by the fact that most of these studies did not adjust for time spent in sedentary behavior. Our findings confirm the new movement guidelines, which not only suggest that youth should move at moderate-to-vigorous intensity, but also meet recommendations on sedentary time and sleep habits [38].

A significant association was found between sedentary behavior time and the likelihood of being MHO in youth only. This result was unexpected, as many studies performed in adults suggested that even when performing more than 150 minutes of MVPA per week, the risk of premature mortality was increased if sitting for six hours or more per day [39, 40]. This difference between youth and adults could potentially be explained by the fact that the peak chronotype is observed in adolescents [41]. Briefly, chronotype is the difference between your awake time and your self-selected awake time. The higher the chronotype, the more one needs to adjust his/her natural circadian rhythm, which can lead to health implications [42, 43]. This recent finding could potentially explain why sedentary behavior impacts more health markers in youth than in adults. To date, no universal consensus has been made in regard to categorizing MHO individuals. Although many different MHO definitions exist [3, 13, 16], some studies used 0 cardiometabolic risk factors, while others used up to ≥3 cardiometabolic risk factors [3, 44]. Therefore, the difference observed in our results compared to others could be explained by the differing MHO definitions used across studies.

Data from our study suggests that any given sedentary period of time that a youth living with obesity spends in a day, whether he/she is physically active, increases the risk of having a poor cardiometabolic risk profile. This is extremely relevant when it comes to determining what to do with this information. As of now, most strategies to improve cardiometabolic risk factor profiles in adolescents focus on increasing physical activity and they fail to do so in the long term [42, 45]. Based on our findings, it seems that reducing sedentary behavior could also be explored as a strategy to improve cardiometabolic health of youth living with obesity. The effect sizes observed in Table 3 are expressed in each reduction of 1 hour of sedentary time, which is not trivial, since the average time spent in sedentary behavior for youth was 476 minutes (7.91 hours). For example, if a youth living with obesity reduces sedentary time by 30 minutes per day, he or she would increase the odds of being MHO by 9.14%. This is alarming since the accelerometry methods did not include nonwear time or sleep time, and almost all youth in the study (99.5%) did at least one 30-minute bout of sedentary behavior and 83.0% did at least one 60-minute bout of sedentary behavior in the evaluated week.

The Canadian Society for Exercise Physiology is now encouraging a comprehensive approach to quantify movements that involve sleep, MVPA, sedentary time, and light activities [38, 46, 47]. This approach is suggested since a recent study that analyzed the 24-Hour Movement Guidelines reported greater cardiometabolic health among youth who met these guidelines compared to those who did not [38]. Our study findings are in accordance with these guidelines that promote looking at activity beyond exercise. Our study adds to these studies by breaking down sedentary behavior time into different bouts and by providing information about sedentary behavior patterns in youth living with obesity, and how they are associated with different cardiometabolic risk factors. Our findings show that any bout of sedentary behavior is associated with the tested cardiometabolic risk factors.

Despite interesting findings, the current study has some limitations that must be highlighted. First, the cross-sectional design of this study limits our ability to make causal interpretations of the data with respect to being considered MHO. Second, cardiorespiratory fitness was not included in our analyses, which might have impacted our results. It is possible that cardiorespiratory fitness would be more associated with cardiometabolic factors in adults rather than MVPA, as previously suggested [48, 49]. However, because of the design of the study, very few individuals had this measure performed in the current study. Despite these limitations, our study is strengthened first by the use of many cardiometabolic risk factors to define MHO, which allows us to capture a broader aspect of health. Second, an objective measure of physical activity and sedentary behavior was used and allowed the quantification of patterns. All of the analyses were performed using weights and clusters to account for the complex survey design of the study. Third, the definition of sedentary time is the number of all wear time minutes with counts below 100. This implies that 60-minute bouts of sedentary time must have at least one count with an intensity greater than 100 somewhere in between the starting and ending epochs. Otherwise, it is counted as nonwear time, instead of sedentary wear time. It can be mentioned that investigating various thresholds for declaring nonwear time and its influence on sedentary behavior patterns is a subject for future research. Fourth, a comprehensive definition of MHO was used, which differs from other literature, and could explain the potential differences between our data and others. Finally, our sample size included a large number of participants in different age groups.

In conclusion, results from our study suggest that sedentary behavior, despite the frequency and duration of the activity, is associated with the likelihood of youth being classified as MHO, while neither sedentary time nor MVPA was associated with the MHO phenotype in adults and older adults. Our results confirm detrimental implications of sedentary time regardless of the physical activity level of the youth, but not in adults and older adults. More studies are needed to investigate the impact of different interventions that aim to reduce sedentary behavior time/patterns and whether this reduction translates into a better cardiometabolic risk factors profile in youth living with obesity.

## Figures and Tables

**Table 1:** General characteristics of non-MHO and MHO participants.

Variable	12–18 years		19–44 years		45–85 years	
	Non-MHO (*N* = 335)	MHO (*N* = 79)	Non-MHO (*N* = 691)	MHO (*N* = 111)	Non-MHO (*N* = 1082)	MHO (*N* = 148)
Age (years)	14.8 ± 2.0	15.2 ± 1.8	32.8 ± 7.5	31.8 ± 7.6	62.1 ± 10.9	60.7 ± 11.0
Male *n* (%)	171 (51.0)	39 (49.4)	308 (44.6)	50 (45.0)	479 (44.3)	59 (39.9)
Ethnicity						
Hispanic *n* (%)	150 (44.8)	21 (26.6)	208 (30.1)	23 (20.7)	217 (20.1)	32 (21.6)
Non-Hispanic Black *n* (%)	65 (19.4)	20 (25.3)	274 (39.7)	50 (45.1)	577 (53.3)	76 (51.4)
Non-Hispanic White *n* (%)	110 (32.8)	38 (48.1)	182 (26.3)	36 (32.4)	265 (24.5)	39 (26.4)
Other *n* (%)	10 (3.0)	0 (0)	27 (3.9)	2 (1.8)	23 (2.1)	1 (0.7)
Waist circumference (cm)	102.1 ± 13.2	98.8 ± 11.8^∗^	111.7 ± 12.9	108.8 ± 11.8^∗^	113.6 ± 10.9	111.5 ± 11.8^∗^
BMI (kg/m^2^)	32.5 ± 5.5	31.8 ± 5.1	35.8 ± 5.4	34.2 ± 4.0^∗^	35.1 ± 4.8	35.0 ± 4.8
BMI *z*-score	2.1 ± 0.3	2.03 ± 0.3	—	—	—	—
Triglycerides (mmol/L)	1.4 ± 0.8	0.8 ± 0.3^∗^	2.3 ± 2.3	1.0 ± 0.3^∗^	2.1 ± 2.1	1.1 ± 0.3^∗^
HDL-cholesterol (mmol/L)	1.1 ± 0.2	1.4 ± 0.3^∗^	1.1 ± 0.3	1.5 ± 0.4^∗^	1.3 ± 0.4	1.5 ± 0.3^∗^
Plasma glucose (mmol/L)	5.3 ± 0.6	4.9 ± 0.4^∗^	5.6 ± 1.4	5.1 ± 0.3^∗^	6.8 ± 2.4	5.1 ± 0.5^∗^
Systolic BP (mmHg)	114.3 ± 10.0	112.1 ± 8.0	120.3 ± 13.3	114.6 ± 10.1^∗^	134.6 ± 20.1	120.6 ± 18.9^∗^
Diastolic BP (mmHg)	59.2 ± 11.0	58.0 ± 11.7	71.8 ± 14.1	68.0 ± 11.1^∗^	72.6 ± 13.3	69.8 ± 9.8

Data are presented as mean ± SD for continuous variables, while categorical variables are presented as *n* (%). MHO = metabolically healthy obese; BMI = body mass index; HDL = high-density lipoprotein. Independent *t*-tests or chi-square tests were performed to test differences between MHO and non-MHO within each age category. ^∗^Significantly different between non-MHO and MHO at *p* < 0.05.

**Table 2:** Sedentary and MVPA bout patterns among non-MHO and MHO individuals.

	12–18 years		19–44 years		45–85 years	
Variable	Non-MHO (*N* = 335)	MHO (*N* = 79)	Non-MHO (*N* = 691)	MHO (*N* = 111)	Non-MHO (*N* = 1082)	MHO (*N* = 148)
1-min. bout (SED-time) *n* (%)	335 (100)	79 (100)	691 (100)	111 (100)	1082 (100)	148 (100)
5-min. bout (SED-time) *n* (%)	335 (100)	79 (100)	691 (100)	111 (100)	1082 (100)	148 (100)
10-min. bout (SED-time) *n* (%)	335 (100)	79 (100)	691 (100)	111 (100)	1082 (100)	148 (100)
30-min. bout (SED-time) *n* (%)	334 (99.3)	78 (98.7)	690 (99.8)	110 (99.1)	1081 (99.9)	148 (100)
60-min. bout (SED-time) *n* (%)	293 (87.5)	62 (78.5)	580 (83.9)	102 (91.9)	1008 (93.2)	138 (93.2)
1-min. bout (MVPA time) *n* (%)	335 (100)	78 (98.7)	689 (99.7)	111 (100)	1037 (95.8)	145 (98.0)
5-min. bout (MVPA time) *n* (%)	314 (93.7)	75 (94.9)	613 (88.7)	107 (96.4)	700 (65.0)	102 (69.0)
10-min. bout (MVPA time) *n* (%)	215 (64.2)	47 (59.5)	307 (44.4)	62 (56.0)	294 (27.2)	53 (36.0)
30-min. bout (MVPA time) *n* (%)	25 (7.5)	7 (9.9)	51 (7.4)	11 (9.9)	67 (6.2)	12 (91.9)
60-min. bout (MVPA time) *n* (%)	1 (<1.0)	0 (<1.0)	2 (<1.0)	2 (<1.0)	4 (<1.0)	1 (<1.0)
1-min. bout (SED-time)	476.0 ± 115.8	473.0 ± 95.5	454.2 ± 119.9	441.4 ± 98.9	519.5 ± 121.7	513.1 ± 127.3
5-min. bout (SED-time)	353.5 ± 121.4	347.2 ± 97.9	330.6 ± 120.9	316.8 ± 97.4	410.9 ± 131.1	398.0 ± 130.4
10-min. bout (SED-time)	254.9 ± 116.0	248.8 ± 92.2	236.6 ± 108.1	224.6 ± 87.2	320.8 ± 129.8	306.1 ± 128.6
30-min. bout (SED-time)	94.8 ± 87.1	87.4 ± 52.1	81.9 ± 59.5	76.1 ± 49.5	137.8 ± 91.2	134.2 ± 90.3
60-min. bout (SED-time)	14.2 ± 66.9	8.22 ± 11.8	10.9 ± 18.8	9.6 ± 17.1	27.9 ± 40.2	29.9 ± 46.7
1-min. bout (MVPA time)	27.8 ± 24.0	25.9 ± 23.2	23.7 ± 20.3	28.6 ± 23.6^∗^	11.3 ± 13.9	12.8 ± 12.6
5-min. bout (MVPA time)	22.4 ± 25.1	20.6 ± 23.3	15.4 ± 19.4	19.8 ± 24.5^∗^	6.8 ± 12.4	7.6 ± 10.0
10-min. bout (MVPA time)	9.5 ± 14.7	8.3 ± 13.5	4.8 ± 10.1	7.0 ± 14.7^∗^	2.8 ± 7.6	2.8 ± 5.6
30-min. bout (MVPA time)	0.8 ± 3.7	0.9 ± 3.2	0.8 ± 3.2	1.7 ± 7.5^∗^	0.8 ± 3.8	1.0 ± 3.8
60-min. bout (MVPA time)	0.1 ± 1.0	0.0 ± 0.0	0.0 ± 0.7	0.5 ± 4.4^∗^	0.0 ± 0.5	0.1 ± 0.7

Data are presented as mean ± SD for continuous variables and *n* (%) for categorical variables. The bouts are expressed as the cumulative bouts of 1-min., 5-min., 10 min., 30-min., and 60-min. bouts. MHO = metabolically healthy obese; min. = minute; SED = sedentary; MVPA = moderate-to-vigorous physical activity. Independent *t*-tests were performed to test differences between MHO and non-MHO within each age category. ^∗^Significantly different between non-MHO and MHO at *p* < 0.05.

**Table 3:** Association between moderate-to-vigorous physical activity and sedentary patterns and the odds of being classified as MHO.

	OR	95% CI	*P* value	
*Youth (12–18 years old)*			
1-min. bout (MVPA)	0.810	(0.338–1.942)	0.636
1-min. bout (sedentary time)	0.824	(0.680–0.999)	0.049
5-min. bout (MVPA)	0.843	(0.362–1.962)	0.691
5-min. bout (sedentary time)	0.837	(0.703–0.997)	0.047
10-min. bout (MVPA)	0.651	(0.163–2.602)	0.543
10-min. bout (sedentary time)	0.847	(0.718–0.999)	0.048
30-min. bout (MVPA)	0.410	(0.005–35.173)	0.694
30-min. bout (sedentary time)	0.819	(0.684–0.980)	0.030
60-min. bout (MVPA)			
60-min. bout (sedentary time)	0.677	(0.479–0.958)	0.030
*Adults (19–45 years old)*			
1-min. bout (MVPA)	1.653	(0.891–3.068)	0.111
1-min. bout (sedentary time)	0.990	(0.875–1.121)	0.877
5-min. bout (MVPA)	1.470	(0.783–2.760)	0.230
5-min. bout (sedentary time)	0.989	(0.886–1.104)	0.840
10-min. bout (MVPA)	1.418	(0.545–3.688)	0.473
10-min. bout (sedentary time)	0.972	(0.873–1.081)	0.595
30-min. bout (MVPA)	3.219	(0.215–48.128)	0.396
30-min. bout (sedentary time)	0.960	(0.832–1.108)	0.577
60-min. bout (MVPA)			
60-min. bout (sedentary time)	1.047	(0.840–1.304)	0.682
*Older adults (45–85 years old)*			
1-min. bout (MVPA)	1.815	(0.900–1.152)	0.182
1-min. bout (sedentary time)	1.018	(0.900–1.152)	0.778
5-min. bout (MVPA)	1.767	(0.728–4.288)	0.208
5-min. bout (sedentary time)	1.019	(0.910–1.140)	0.748
10-min. bout (MVPA)	1.070	(0.226–5.069)	0.932
10-min. bout (sedentary time)	0.992	(0.894–1.100)	0.873
30-min. bout (MVPA)	0.850	(0.042–17.253)	0.916
30-min. bout (sedentary time)	0.952	(0.852–1.063)	0.380
60-min. bout (MVPA)			
60-min. bout (sedentary time)	0.968	(0.828–1.131)	0.678

Data are presented as odds ratio (OR) and 95% confidence interval (CI). OR is expressed in hours. The youth models were adjusted for BMI *z*-score, age, sex, ethnicity, family income, time (1-min. bout) spent in a sedentary state, and MVPA. Adults and older adults models were adjusted for BMI, age, sex, ethnicity, education, family income, time (1-min. bout) spent in a sedentary state, and MVPA.
